# Barriers to Mental Illness Treatment in Saudi Arabia: A Population-Based Cross-Sectional Study

**DOI:** 10.7759/cureus.53797

**Published:** 2024-02-07

**Authors:** Ahmed A Mohamed, Sufyan M Alomair, Abdulrahman A Alnijadi, Fatimatuzzahra Abd Aziz, Abdulaziz S Almulhim, Mohamed A Hammad, Promise M Emeka

**Affiliations:** 1 Clinical Pharmacy, College of Clinical Pharmacy, King Faisal University, Al-Ahsa, SAU; 2 Clinical Pharmacy, School of Pharmaceutical Sciences, Universiti Sains Malaysia, Penang, MYS; 3 Pharmacy Practice, College of Clinical Pharmacy, King Faisal University, Al-Ahsa, SAU; 4 Clinical Pharmacy, Al-Baha University, Al-Baha, SAU; 5 Pharmaceutical Sciences, College of Clinical Pharmacy, King Faisal University, Al-Ahsa, SAU

**Keywords:** obstacles, consultation, stigma, barriers, mental illness

## Abstract

Background: Mental illness is a disorder that can cause impairment and disability, affecting mood, thinking, and behavior; therefore, early intervention will reduce morbidity. This study aims to evaluate all the personal, family, societal, and medical barriers that prevent mental health patients from seeking consultation and treatment.

Methods: In Saudi Arabia, a cross-sectional study was conducted on 463 individuals aged 18 and above. Data were collected by face-to-face interviews using a validated questionnaire, which consisted of two parts. The first part included sociodemographic data, while the second part contained subsections of society/family, personal, and medical barriers.

Results: The results showed that 379 (81.9%) indicated that society and family barriers impacted them, whereas 325 (70.3%) believed that personal barriers hindered seeking help. However, 294 (63.5%) opted for medical barriers as a hindrance. Regarding the highest barriers, 120 of the total respondents (25.9%) saw psychiatric illness as a source of shame and stigma, 166 respondents (35.9%) said that the psychiatric patient is seen as crazy, 159 of them (34.3%) believed it is tough for anyone to talk about their feelings and emotions and 183 respondent (39.5%) feared that psychiatric illness would decrease the chance of marriage to the appropriate person. Our findings also indicated a low trust in hospital treatment, hence a loss of confidence in using medications.

Conclusion: The findings of this study indicate that societal stigma is the most common barrier preventing people from seeking mental health consultation. Many barriers differ significantly between males and females.

## Introduction

Mental illness has many consequences on different stages of life, and it is a leading cause of disability, a cause of higher rates of drug abuse and mortality secondary to suicide. In addition, it interferes with normal social development and delays the educational trajectory, with a growing worldwide economic burden [1-4]. In addition, mental disorders impact the quality of life, the level of functioning and interfere with several socio-economic factors [5,6]. Seeking psychiatric consultation is the first step in dealing with mental illness and helps avoid its negative impact on patients' lives. However, unlike other medical conditions, living with mental health illness has several challenges. The chief among those challenges is the delay in seeking medical consultation, which has been reported to affect the treatment outcome negatively [7], suggesting that it can increase the morbidity and mortality rates, prepone the onset of mental and physical comorbid conditions, and increase the likelihood of using life-threatening and life-altering illegal self-treatments. It is also significantly associated with increasing the complexity of the healing process [8].

Many barriers were shown to prevent help-seeking. One of the main barriers is stigma. Stigma is described as the fear of being socially sanctioned or discredited that can lead to the concealment or prevention of certain acts or behaviors [9]. It was reported to be the main stumbling block preventing patients from seeking mental health consultation, hence creating a gap between service and treatment [10,11]. The second most reported barrier is the mistaken perceptions and beliefs about the nature of mental illness, the appropriate management methods, and dealing with mentally ill patients [12]. Additional barriers include low perceived need and patients' attitudes to the disease [13-15]. Besides inappropriate behaviors toward the person with mental illness, the negative social perception of mental illness is also a significant barrier preventing help-seeking. In consequence, patients prefer to suffer in silence [16-18]. Another factor that plays a crucial role in preventing anyone from reporting that they need consultation is the fear of hospital admission into the psychiatric ward due to losing confidence in the level of mental health care services provided [19]. Therefore, these factors have impacted the treatment course and impaired engagement with the medical services and the community. It is envisaged that this behavior will ultimately increase the likelihood of therapy failure and adverse outcomes [20]. The myths of peoples' beliefs concerning mental health medications suggest that they cause addiction, lose their effects over time, and even worsen their case [21]. These myths, therefore, form barriers that prevent people from seeking medical help even when they need it. However, misconceptions abound in the Saudi population, area of the present study, about the causes and nature of mental illness. They believe it is caused by envy, evil eye, and jinn. In these cases, they take the patients to faith healers to solve this problem [22].

The abovementioned barriers have been reported in literature among many societies [23-25]. This, in turn, increases the suffering of many people who need assistance; nevertheless, they prefer to keep silent and avoid discussing their mental health problems or seeking professional medical consultation. This attitude is common among many populations; therefore, it can be potentially consequential in social and economic outcomes. Thus increasing the burden on the governments and community in general [26,27]. Therefore, the first step in solving these problems is to evaluate the general population's attitude/view of these barriers and their effect on preventing patients from seeking mental illness treatment [28]. The evaluation process can guide the government to provide adequate healthcare support for mental health patients suffering silently. To the best of our knowledge and after a careful literature search so far in the present studies done in Saudi Arabia, many studies focus on stigmatizing beliefs in the Saudi population [29,30], with a study assessing the barriers among hospitalized psychiatric patients [14], while no population-based assessment has been done before to evaluate the knowledge and attitudes toward mental illness and assess the potential barriers that may prevent those who need professional mental illness counseling seek help. The primary objective of this study is to evaluate the personal, family, societal, and medical barriers that prevent mental health patients from seeking consultation and treatment. The secondary objective is to find any gender variation in the study barriers.

## Materials and methods

Study design

A population-based cross-sectional survey was conducted from January to March 2020 in Saudi Arabia. The participants were invited to respond to a survey in a face-to-face interview.

Study population

To ensure that the study sample was representative of the eligible population, convenience sampling was used to select eligible individuals who met the study's inclusion criteria based on their accessibility and willingness to participate. Participants were eligible for this study if they were 18 years or older, were residents of Saudi Arabia, were not diagnosed with any psychiatric illness diseases, could participate in a face-to-face interview, and signed the informed consent. According to the latest World Population Review report, Saudi Arabia's total population is 36,769,366, and 25,308,476 people are over 18 [31]. Therefore, based on the population proportion formula (formula n = N*x/((N - 1)*E2 + x) [32], the required sample size is calculated to be 385. Participants who did not agree to sign the informed consent were excluded.

Instrument for data collection

The tool used to collect the data was designed based on 30-item barriers used for the assessment adopted from the Barriers to Access to Care Evaluation (BACE) scale [33], a reliable and validated scale developed to assess the different obstacles that prevent adults from seeking mental health consultations and care. In the present study,

We analyzed the 30-item scale and modified some items to increase the feasibility of using this tool with the Saudi population. The initial version was translated to Arabic by academic English linguistic translation to the Arabic language. Then, this Arabic version was sent to a native English healthcare provider with an excellent Arabic background to translate this version back to English. Then, both versions, Arabic and English, were revised to confirm non-significant differences. The final Arabic version was sent to eight psychiatrists, clinical pharmacists, and psychotherapists. Their feedback validated the questionnaire items to measure our research target and confirm face and content validity. All the comments from the reviewers were addressed, and the research team reviewed and approved the final version. This process adhered to the questionnaire instrument guidelines for translation, adaptation, and validation [34]. The research ethics committee at the Deanship of Scientific Research at King Faisal University approved the study protocol (KFU-REC/20201001).

The final form of the study tool consisted of two parts. The first part consisted of the informed consent and the participant's demographics (Gender, Age, Social Status, Level of Education, and Occupation). The second part assessed the barriers preventing people from seeking mental health consultation and treatment, consisting of 29 questions, each rated with a 5-point Likert scale response (Strongly Agree, Agree, Neutral, Disagree, and Strongly Disagree). Hence, this part was subdivided into three subsections; the first subsection evaluates the obstacles related to family and society as stigma and how society judges mentally ill patients. The second subsection was to assess personal feelings and attitudes preventing seeking help. The last part was to evaluate the misconceptions about mental health illness and the treatment pathways. In all sections, For each barrier, we calculated the percentage of any degree of acceptance and then ranked the barriers as all, and in each section, we tried to find the most prevalent barriers between the study participants. In our research, we divided the 30 items from the validated BACE survey into three sections only to make the questionnaire easier for the participants and for more profound interpretation of results. Yet, we calculated the questionnaire’s consistency based on the original validated BACE survey. 

Data analysis

Statistical analysis was performed using IBM SPSS Statistics for Windows, version 26.0 (IBM Corp., Armonk, NY, USA). The difference in response between males and females was calculated using the Mann-Whitney U test for each item. We set the level of statistical significance at 0.05. Also, to confirm the internal consistency of the Arabic version, the Cronbach α coefficient was calculated on 20 randomly selected responses, with a value of 0.7 but not more than 0.9. The analysis of the responses to the barriers mentioned in the questionnaire was based on the level of agreement of the respondents [33]. The internal consistency results indicated a Cronbach's α value of 0.862, which confirmed the questionnaire's reliability.

## Results

Participant demographics

We interviewed a total of 463 individuals. The participants' demographics are represented in Table 1 distributed by gender for each characteristic and as a total. The study sample comprised 186 males (40.2%) and females represented 277 (59.8%). Age group distribution indicated that 178 (38.5%) of participants were between 26 and 40 years old, reflecting the maximum number of participants. The participants' social status was divided almost equally between single and married, with 226 (48.8%) and 222 (48%). Regarding the level of education, the largest portion was the university graduates, who comprised 313 (67.6%) of the participants. Students represent 144 (31%) of the participants, and 206 (44.5%) were employed. Among the females, 52 (11.1%) were housewives. 

**Table 1:** Demographics information of respondents

Demographics		Respondents gender distribution				Total No of respondents	
		Male		Female			
		N	(%)	N	(%)	N	%
Gender		186	40.2	277	59.8	463	100
Age (years)	18-25	62	13.4	109	23.5	171	36.9
	26-40	92	19.9	86	18.6	178	38.5
	41-60	24	5.2	71	15.3	95	20.5
	More than 60	8	1.7	11	2.4	19	4.1
Social Status	Single	105	22.7	121	26.1	226	48.8
	Married	79	17.1	143	30.9	222	48
	Divorced	2	0.4	13	2.8	15	3.2
Level of Education	Uneducated	1	0.2	0	0	1	0.2
	High school	17	3.7	53	11.4	70	15.1
	University degree	149	32.2	164	35.4	313	67.6
	Higher degree	19	4.1	60	13.0	79	17.1
Occupation	Student	51	11.0	93	20.1	144	31.1
	Employed	118	25.5	88	19.0	206	44.5
	Unemployed	11	2.4	19	4.1	30	6.5
	Housewife	-	-	52	11.2	52	11.2
	High qualification but unemployed	6	1.3	25	5.4	31	6.7

Barriers to mental help-seeking analysis

The participants' responses for all barriers were represented in two levels of agreement (Table 2). The first one, entitled “Higher level of agreement,” included the respondents who selected “Strongly agree” only. The second one, entitled “Any level of agreement” included the total number of participants who chose either “Strongly agree,” “Agree,” or “Neutral.” Both levels of agreement were represented as numbers and percentages from the total number of participants. The barriers were compared according to the number of respondents of “Strongly agree” each had. They were arranged in descending order, where No. 1 was given to the barrier with the highest number of participants choosing “Strongly Agree”.

**Table 2:** Participants' responses for barriers to mental help-seeking *The percentage of the number of participants who chose “Strongly agree” to the total number of participants (463) **The percentage of the number of participants who chose “Strongly agree + Agree + Neutral” to the total number of participants (463) ***The order of the barrier after arranging them in descending order according to the number of respondents of “Strongly agree”, where No. 1 was given to the barrier, with the highest number of participants choosing “Strongly agree”.

Barriers	Higher level of agreement (Strongly agree) (n)	Higher level of agreement (Strongly agree) (%) *	Agree with any level of agreement (Strongly agree+ Agree+ Neutral) (n)	Agree with any level of agreement (Strongly agree+ Agree+ Neutral)(%) **	Order of the barrier ***
Barriers Related to Family and Society (F&S)					
The psychiatric illness is a source of shame and stigma (F&S 1)	120	25.9	361	78.0	8
The psychiatric patient will become lonely and lose all of the social relations (F&S 2)	112	24.2	398	86.0	11
Having a psychiatric illness will decrease the chance of getting an excellent job opportunity (F&S 3)	145	31.3	403	87.0	5
Having a psychiatric illness will decrease the chance of marriage in an appropriate person (F&S 4)	183	39.5	423	91.4	1
The psychiatric patient is seen as crazy (F&S 5)	166	35.85	379	81.9	2
The Financial and psychological burden of psychiatric treatment is too expensive (F&S 6)	113	24.4	376	81.2	10
Families deny sending their children to a psychiatrist due to family ego (F&S 7)	137	29.6	399	86.2	6
Children who have psychiatric or behavioral disturbances, if sent to the mental hospital, their condition will worsen (F&S 8)	89	19.2	353	76.2	14
The psychiatric patient is regarded as a weak person in society (F&S 9)	109	23.5	396	85.5	13
The psychiatric patient is a source of mockery (F&S 10)	110	23.8	362	78.2	12
The psychiatric patient is unable to face life challenges (F&S 11)	81	17.5	322	69.5	15
Mean (SD)		26.80 (6.39)		81.92 (6.17)	
Personal Barriers					
Feeling embarrassed or inferior (P 1)	155	33.5	408	88.1	4
Difficulty in having a sick leave from work or school (P 2)	80	17.3	359	77.5	16
Personal responsibilities prevent seeking mental health advice (e.g., Having children) (P 3)	77	16.6	363	78.4	17
It is not clear where to go to seek mental health consultation (P 4)	128	27.6	356	76.9	7
Psychiatric disorders are due to the evil eye and envy (P 5)	71	15.3	285	61.6	20
The belief that having mental problems is due to lose of or weak faith (P 6)	44	9.5	227	49.0	22
Anyone could easily control the psychiatric illness by themselves, and the problem could be solved without any support (P 7)	32	6.9	201	43.4	26
It is tough for anyone to talk about their feelings and emotions (P 8)	159	34.3	416	89.8	3
Admitting to having a mental illness will increase the feeling of guilt (P 9)	76	16.4	314	67.8	18
Mean (SD)		19.71 (9.87)		70.28 (16.17)	
Medical Barriers					
The person admitted to the mental health hospitals will be treated as an outcast (M 1)	31	6.7	256	55.3	28
The psychiatric patients' condition will worsen when they are admitted to the mental health hospitals (M 2)	25	5.4	266	57.5	29
The only thing that mental health hospital provides is the medications (M 3)	54	11.7	279	60.3	21
The admission to a mental health hospital is terrifying (M 4)	120	25.9	390	84.2	9
The psychiatric medications cause addiction (M 5)	72	15.6	298	64.4	19
The psychiatric medication side effects are intolerable (M 6)	44	9.5	357	77.1	23
Whoever starts using psychotropic medication will use it for life (M 7)	32	6.9	260	56.2	27
The effectiveness of the psychotropic medication is in doubt (M 8)	36	7.8	266	57.5	25
I saw previous unsuccessful experiences of acquaintances who received psychotherapy (M 9)	44	9.5	276	59.6	24
Mean (SD)		11 (6.37)		63.57 (10.2)	

The barrier given No. 1 was concerning the fear that seeking mental illness treatment would interfere with a person's social life, and decrease their ability to get married. One hundred eighty-three participants (39.5%) selected that they “strongly agree” with this barrier. The second-ranked barrier was the society's stigma that considered a mentally ill patient a crazy person, with 166 (35.9%) reporting that.

Barriers Related to Family and Society

**Figure 1:**
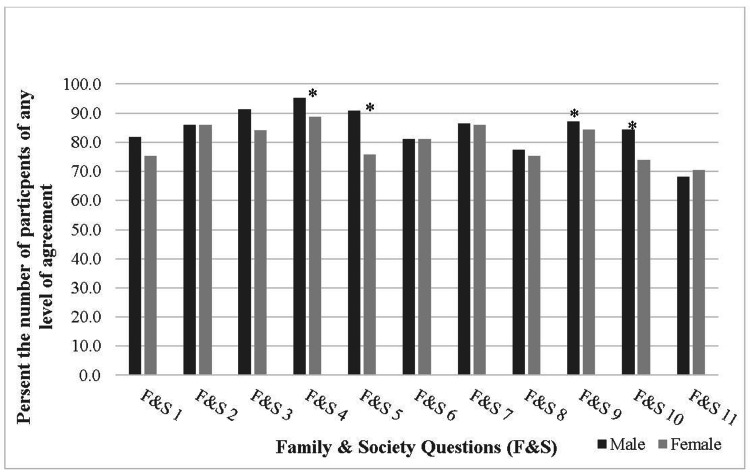
Barriers related to family and society *Indicates significant result

The “Agree with any level of agreement” was the highest in this section compared to others, with an average agreement of 81.9% (±6.17). The two highest-ranked barriers were both in this section. These were the fear of losing the chance of getting married when the person was diagnosed with mental illness and the worry of being called crazy by society (P<0.05) and (P<0.001), respectively (Figure 1). The male respondents reported a higher agreement level in those two points compared to females. Two additional items indicated a significantly higher level of agreement in males than females (P<0.05) for both. These were the barriers entitled: "The psychiatric patient is regarded as a weak person in society" and "The psychiatric patient is a source of mockery". They were ranked 13 and 12, respectively.

Personal Barriers

**Figure 2:**
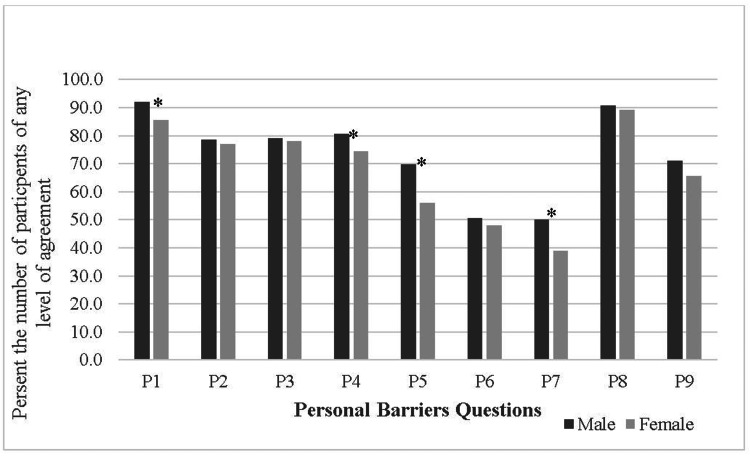
Personal barriers *Indicates significant result

The average of respondents' “Agree with any level of agreement” in this part was 70.3% (±16.27). Among the personal barriers, "finding it tough for anyone to talk about his/her feelings and emotions" was found to be the third-ranked item, while "feeling embarrassed or inferior" was ranked fourth and was significantly higher in males than females (P<0.05) (Figure 2). Other barriers that were found to be significantly higher in males than females also included: "It is not clear where to go to seek mental health consultation" (P<0.001), "Psychiatric disorders are due to evil eye and envy" (P<0.001), and "Anyone could easily control the psychiatric illness by him/herself, and the problem could be solved without any support" (P<0.05).

Medical Barriers 

**Figure 3:**
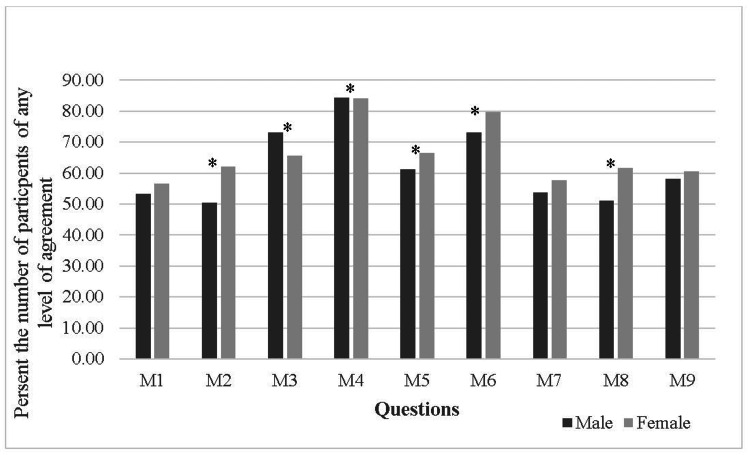
Medical barriers *Indicates significant result

The total respondents' “Agree with any level of agreement” on average was 63.5% (±10.2) for medically related barriers. The barrier of fear of going to the hospital was ranked ninth according to the highest level of agreement, which was the highest rank given to any of the medical barriers. Unlike other barriers, most medical barriers were significantly higher in females than males. For example, they expressed phobia about going to the hospital (P<0.001) and believed that the hospital would not benefit them except for providing medications (P=0.001). Also, females reported that hospital admission would worsen their condition (P<0.05) (Figure 3).

## Discussion

The present study has shown that stigmatization from society and family challenges are the significant barriers that prevent most people from seeking mental health consultation. This is in concordance with previously published studies that documented that social stigma is the main barrier preventing patients from seeking help [11,35]. Hence, stigma creates a gap between service and treatment [10,11]. Individuals who have a mental illness do not only deal with the burden and disability brought on by their symptoms but also deal with the stigma attached to their disease in society [29]. 

Concerning gender differences, barriers related to family, society, and personal issues were significant, with a higher level of agreement with male respondents. Male participants appeared to be more concerned about society's and the family's view of the person, which could impact the individual's chance of getting married or having a good job opportunity. The gender difference concerning mental health stigma found in previous studies was not in consensus. Some studies found that females were more concerned about stigma [36,37], which impacts their tendency to seek medical help, while other studies found this factor to be equal among both genders [38,39]. These findings were contrary to the observation of the present study, indicating that male subjects were more particular about social stigma being a barrier to seeking medical advice.

Another common factor that most studies reported as a barrier is shame, which prevents patients from talking freely about mental health disorders [40]. In our study, the difficulty of talking freely about the disease was ranked third among the barriers. This finding is consistent with results found in a systematic review that reported shame as one of the long-term mental illness difficulties [41].

Also, cultural beliefs about the causes and nature of mental illness impacted mental help-seeking, as many participants believed psychiatric disorders are due to the evil eye and envy. These findings were confirmed by another study performed on the Saudi population, which showed that patients were usually taken to faith healers rather than mental health institutions [22]. This barrier prevents people from seeking medical help even when they need it.

One of our study's critical findings is female participants' greater fear of medical barriers than their fear of society, family, and personal obstacles. Females see admission to the psychiatric hospital as a terrifying experience that will lead to no improvement. They reported that the hospital only gives many ineffective medications that can cause addiction and have not been able to control mental illness. These findings indicate a low level of trust in in-hospital treatment of mental illness among female participants. So, loss of confidence in the therapeutic pathway will affect the compliance of the patients [42]. These results agree with a recent review, which concluded that the level of female adherence to psychiatric medications is lower. However, others have reported that male patients have a higher chance of non-adherence [43].

In general, the prevalence of psychiatric illness is higher in females than in males, reaching more than two-fold in some diseases such as depression and anxiety [44]. This means that females have more need for psychiatric medical care services. The problem arises when the patient has fears about the medical care settings. This fear can be a barrier preventing the patient from seeking the proper treatment to avoid facing his/her anxiety, leading to a lower level of disease control [45], increasing the complexity of the disease [46,47], and need more complicated and long-term treatment plans [44,48,49]. Unfortunately, we found that the medical barriers, which included fear of hospitalization and mistrust in the service provided, were significantly higher in females. We are concerned that this might lead to more medical care avoidance among females and, hence a lower rate of psychiatric illness management.

Our study has some limitations that may impact the generalizability of the results. The sample size needs to be increased and cover more social categories not represented in our study population. One of the limitations of our study is that most respondents belong to similar social groups, mostly university graduates (almost 50% of the respondents). Our study does not represent people living in remote areas who tend to have a lower level of healthcare facilities, as we could not reach these areas during data collection. Further studies are required with larger sample sizes that fully represent the different socio-economic populations within the society. 

The findings of our study clarified the different obstacles that prevent many people from seeking mental health services. This understanding will enable the healthcare authorities to design targeted education programs, patient approach strategies, and campaigns, which, in turn, will improve the quality of the mental healthcare systems.

The results of this study can guide policy-makers in developing rules and regulations that ensure an effective and safe healthcare service that people feel comfortable seeking. During our interview, many participants -especially females- expressed their fear of seeking mental health care due to past negative experiences for them or their relatives. Such findings can direct health care management towards changing the profile of mental health care services presented specifically to females and providing a service that focuses on the emotional and psychological aspects of the patient, not just the medical aspect. In other words, health care providers need to be aware of the factors within the institutions that push people away or even frighten them to the extent that they prefer to suffer silently about their mental illness rather than seek a mental health care service.

## Conclusions

This study showed that society and family barriers were significant obstacles to seeking help for mental health in the area of study. In addition, while males saw society and family as their primary barrier, females, on the other hand, indicated that medical barriers were their issue. They further expressed the fear that medications did not help, caused side effects, and could require a life-long commitment to treatment. The financial impact of mental disease in Saudi Arabia can be minimized by increasing access to care and lowering the stigma around mental health. Therefore, educating the population and making them aware that the government has put protective guidelines and treatments will allay those fears and create a mentally healthy society. When educating the population, it is important to put the gender difference into consideration in order to address each gender’s concerns effectively.
